# Radiation Induced Apoptosis of Murine Bone Marrow Cells Is Independent of Early Growth Response 1 (EGR1)

**DOI:** 10.1371/journal.pone.0169767

**Published:** 2017-01-12

**Authors:** Karine Z. Oben, Beth W. Gachuki, Sara S. Alhakeem, Mary K. McKenna, Ying Liang, Daret K. St. Clair, Vivek M. Rangnekar, Subbarao Bondada

**Affiliations:** 1 Department of Microbiology, Immunology and Molecular Genetics, University of Kentucky, Lexington, Kentucky, United States of America; 2 Department of Toxicology and Cancer Biology, University of Kentucky, Lexington, Kentucky, United States of America; 3 Department of Internal Medicine, University of Kentucky, Lexington, Kentucky, United States of America; 4 Department of Radiation Medicine, University of Kentucky, Lexington, Kentucky, United States of America; 5 Markey Cancer Center, University of Kentucky, Lexington, Kentucky, United States of America; University of South Alabama Mitchell Cancer Institute, UNITED STATES

## Abstract

An understanding of how each individual 5q chromosome critical deleted region (CDR) gene contributes to malignant transformation would foster the development of much needed targeted therapies for the treatment of therapy related myeloid neoplasms (t-MNs). Early Growth Response 1 (EGR1) is a key transcriptional regulator of myeloid differentiation located within the 5q chromosome CDR that has been shown to regulate HSC (hematopoietic stem cell) quiescence as well as the master regulator of apoptosis—p53. Since resistance to apoptosis is a hallmark of malignant transformation, we investigated the role of EGR1 in apoptosis of bone marrow cells; a cell population from which myeloid malignancies arise. We evaluated radiation induced apoptosis of *Egr1*^*+/+*^ and *Egr1*^*-/-*^ bone marrow cells in vitro and in vivo. EGR1 is not required for radiation induced apoptosis of murine bone marrow cells. Neither p53 mRNA (messenger RNA) nor protein expression is regulated by EGR1 in these cells. Radiation induced apoptosis of bone marrow cells by double strand DNA breaks induced p53 activation. These results suggest EGR1 dependent signaling mechanisms do not contribute to aberrant apoptosis of malignant cells in myeloid malignancies.

## Introduction

Myeloid malignancies are clonal diseases which arise from hematopoietic stem or progenitor cells [1]. Based on the reported cases, it is estimated that there will be 28,000 new cases and 11,000 deaths in the United States this year due to myeloid malignancies [2]. Several genetic alterations have been identified in myeloid neoplasms (MN) but our understanding of their individual effects and how they each contribute to disease development is still limited [1]. Such understanding will facilitate separation of driver mutations from the plethora of background mutations, hence enhancing our ability to develop targeted treatments as was demonstrated by the identification and characterization of the break point cluster region-abelson (Bcr-Abl) driver genetic alteration in chronic myeloid leukemia [3]. Deletions in chromosome 5 (del (5q)) or complete loss of the entire chromosome 5 (-5) is one of the most common cytogenetic abnormalities observed in therapy related myeloid neoplasms (t-MNs) [4, 5]. t-MNs are thought to occur as a late complication of cytotoxic therapy (radiotherapy and or chemotherapy), typically for a primary malignancy [6]. Though the 5q chromosomal deletions that occur in t-MNs are typically very large, uncharacteristically smaller deletions occur in a few patients [7, 8]. These uniquely smaller deletions facilitated delineation of the critical or common deleted region (CDR) by gene mapping [4, 7, 8].

A hallmark of malignant cells is the evasion of tumor suppressors [9]. Several genetic mechanisms mediate malignant cell evasion of tumor suppressors including deletion of a genetic locus or complete chromosome loss [10]. According to Knudson’s 'two-hit' hypothesis, both alleles of a tumor suppressor gene have to be mutated in order for malignancy to occur [11]. The deletions in chromosome 5 observed in myeloid malignancies suggest the likelihood that one or more tumor suppressor genes may be present in the CDR [12, 13]. The genes located in the CDR of chromosome 5q have been identified [4, 14] but they do not conform to Knudson’s 'two-hit' model of tumor suppressor genes as there are no known genetic lesions on the undeleted allele in t-MNs [4, 15, 16]. Growing evidence supports the possibility that haploinsufficiency of one or more genes can promote malignancy [17–19]. Therefore, it is of paramount importance to delineate the role of individual 5q chromosome CDR genes in malignant transformation.

Early Growth Response 1(*EGR1*) is a putative tumor suppressor gene located in the CDR [4, 7]. EGR1 is a zinc finger DNA-binding protein that is rapidly induced, within minutes, by mitogens in cultured cells [20, 21]. EGR1 has been shown to modulate murine hematopoietic stem cell (HSC) proliferation and mobilization by promoting HSC quiescence and retention in the bone marrow niche [22]. It is a key transcriptional regulator of myeloid cell differentiation [23, 24], therefore a good tumor suppressor candidate gene in t-MNs. In fact, there is evidence to suggest it directly regulates well known tumor suppressors TGFβ1, PTEN, p53 and fibronectin [25]. EGR1 positively regulates the expression of macrophage specific genes while repressing neutrophil specific genes [24]. Interestingly, haploinsufficiency of EGR1 increased the frequency of myeloproliferative disorder (MPD) and decreased latency in mice treated with a DNA alkylating agent-N-ethyl-nitrosourea (ENU), to induce secondary mutations [26]. These studies did not elucidate the mechanism by which loss of EGR1 promotes malignant transformation in ENU treated mice. One other mechanism by which EGR1 could enhance MPD is through its effects on the apoptosis pathway in the hematopoietic stem or progenitor cell populations.

Resistance to apoptosis is another hallmark of malignant transformation [9]. Studies defining the role of EGR1 in apoptosis are conflicting. EGR1 has a proapoptotic function in murine intestine and embryonic fibroblasts owing to its ability to stabilize p53 protein [27, 28]. The proapoptotic effect of EGR1 has also been observed in patients with advanced laryngeal and hypopharyngeal squamous cell carcinoma (LHSCC) receiving chemoradiation therapy [29]. In these patients, high EGR1 expression significantly correlated with therapy response and survival rate. Conversely, EGR1 rescued pancreatic β-cells from apoptosis[30] and counteracted p53-dependent apoptosis in the human fibrosarcoma cell line HT1080 [31]. Differential regulation of EGR1 downstream targets, p300 and CREB binding protein (CBP) in different cell types could explain the opposing effects of EGR1 on apoptotic signals [32]. Cells in which EGR1 represses p300/CBP transcription receive pro-apoptotic signals while cells which increase EGR1 driven p300/CBP expression receive anti-apoptotic signals. The effect of EGR1 on apoptosis, pro- or anti, appears to be p53 dependent and cell specific [27, 28, 31, 33, 34]. The role of EGR1 in the apoptotic response of primary bone marrow cells has not been elucidated. This could have implications in the leukemogenesis of myeloid malignancies with chromosome 5 genetic alterations. The purpose of this study, therefore, was to determine if EGR1 plays a role in radiation induced apoptosis of bone marrow cells, and to determine whether the EGR1-p53 pathway is activated to mediate this apoptotic response. Our data suggest that EGR1 does not play a significant role in radiation-induced apoptosis of murine bone marrow cells. Interestingly, we show that radiation induces upregulation of p53 protein levels equally well in EGR1 deficient and sufficient bone marrow cells. It is likely that radiation induced activation of the double strand DNA break (DSB) repair pathway induces p53 in bone marrow cells, which may be responsible for their apoptosis response.

## Materials and Methods

### Reagents

Recombinant mouse stem-cell factor (mSCF)–(455-MC-050), mouse interleukin-3 (mIL-3)–(403-ML-010), and human interleukin-6 (hIL-6)–(206-IL-050) were purchased from R & D Systems (Minneapolis, MN). Biotin conjugated rat anti-CD45R/B220 (553086), anti-CD11b (553309), anti-Gr-1 (553125), anti-CD8a (5532029), anti-Ter-119 (553672), anti-CD5 (553019); streptavidin APC CY7 (554063) and anti c-KIT-APC (553356) were purchased from BD PharMingen (San Diego, CA). Anti-Sca-1-PB (122520) was purchased from BioLegend (San Diego, CA). Dynabeads sheep anti-rat IgG (11035) was obtained from Life Technologies (Carlsbad, CA). Annexin V apoptosis detection kit (88-8103-72) was purchased from eBioscience (San Diego, CA). Antibodies to p53 (2425S), cleaved caspase-3 (9661S), P-Chk2 (2661T) and γ-H2AX (Ser139) (9718P) were purchased from Cell Signaling Technology (Danvers, Massachusetts). Alexa Fluor 488 conjugated anti-γ-H2AX (Ser139) (9719) and rabbit IgG (4340) were also purchased from Cell Signaling Technology. DyLight 488 conjugated AffiniPure F(ab')2 goat anti-rabbit antibody was from Jackson Immunoresearch (#111–486–046). Peroxidase coupled goat anti-rabbit (SC-2004) and anti-mouse (SC-2005) Ig secondary antibodies were obtained from Santa Cruz Biotechnology (Santa Cruz, CA).

### Mice

*Egr-1*^*+/-*^ breeding pairs were obtained from The Jackson Laboratories (Bar Harbor, ME) and bred at the University of Kentucky’s Division of Laboratory Animal Resources (DLAR) AAALAC certified animal facility. The Jackson Laboratories genotyping protocol by polymerase chain reaction (PCR) was used to type pups. *Egr1*^*+/+*^ and *Egr1*^*-/-*^ littermates were used for the study. The primer sequences for genotyping were as follows: WT forward, 5’- AACCGGCCCAGCAAGACACC-3’; KO forward, 5’-CTCGTGCTTTACGGTATCGC-3’; common reverse primer, 5’-GGGCACAGGGGATGGGAATG-3’ (IDT Technologies Inc. Coralville, Iowa). Animals had free access to food and water, and were housed with a 12-hour light–dark cycle and constant temperature. Mice were monitored by body posture and activity level [35] daily for a week after irradiation and 3X a week thereafter until the experiment was terminated. Euthanasia was performed by carbon dioxide and cervical dislocation. The University of Kentucky’s Institutional Animal Care and Use Committee (IACUC) approved these studies.

### Isolation of bone marrow mononuclear cells (BM-MNCs), enrichment of LIN-ve cells, normal B cells and cell culture

Tibiae and femora were harvested from mice (15–20 WT and *Egr1*^*-/-*^). The bones were flushed with a 26G syringe in HBSS containing 2% fetal bovine serum (FBS) (Atlanta Biological Systems). Ficoll-paque^TM^ plus (GE Healthcare 17-1440-03) was used to isolate BM-MNCs from pooled bone marrow cells. BM-MNCs were incubated with normal rat IgG (10μg/1 × 10^6^ cells) at 4°C for 15 min to block Fcγ receptors. The cells were then labeled with biotin coupled rat anti-mouse lineage specific antibodies to CD11b (Mac-1), B220, Gr-1, CD8α, Ter-119 and CD5. Labeled BM-MNCs were incubated with anti-rat IgG coupled magnetic beads at a bead to cell ratio of ~ 3:1. Labeled mature lymphoid and myeloid cells bound to beads were depleted three times by magnetic field separation. Lineage negative (LIN-ve) enriched BM-MNCs that did not bind the beads were washed 2X in HBSS-2% FBS and suspended at 1 × 10^6^/ml in complete medium (IMDM supplemented with 15% FBS (HyClone) and 2% Penicillin-Streptomycin-Amphotericin B (Lonza)– 200 units/ml). LIN-ve enriched BM-MNCs (1 × 10^6^/ml) were stimulated with cytokines (mSCF-50ng/ml, mIL3-10ng/ml, hIL6-10ng/ml) in complete IMDM for 2 days to induce proliferation. Normal spleen B cells were prepared by T cell depletion as previously reported [36].

### Irradiation of BM-MNCs and mice

LIN-ve enriched BM-MNCs (1 × 10^6^/ml) suspended in complete medium + cytokines were exposed to 2 Gy or 6 Gy irradiation in a Mark I-68 ^137^Cesium γ-irradiator (J.L Shepherd and Associates). Mice were exposed to a 6.5 Gy sub-lethal dose of irradiation. Cells and mice were irradiated on a rotating platform.

### Apoptosis assays

24 Hrs after irradiation, LIN-ve enriched BM-MNCs were incubated with normal rat IgG (10μg/1 × 10^6^ cells) at 4°C for 15 min to block Fcγ receptors. The cells were then stained with c-KIT-APC, Sca-1-PB and streptavidin APC CY7 antibodies for 30 min at 4°C in the dark. Stained cells were washed 2X with fluorescent activated cell sorter (FACS) buffer (1X phosphate buffered saline without calcium or magnesium, supplemented with 25mM Hepes, 5mM EDTA and 1% FBS) and stained with annexin-V-PE CY7 following manufacturer’s protocol. Positively stained cells were detected by the BD LSRII flow cytometer and the data was analyzed by the FlowJo (Ashland, OR) single cell analysis software.

The apoptosis assay by intracellular staining for activated or cleaved form of caspase-3 was performed according to the Cell Signaling Technology protocol. BM-MNCs were stained 6 Hrs after exposure to 6 Gy irradiation. The recommended antibody dilution for flow cytometry (1:800) was used. Cells were then stained with secondary DyLight 488 conjugated AffiniPure F(ab')2 goat anti-rabbit antibody (1:200) for 1hr in the dark. After washing 2X with FACS buffer, positively stained cells were detected by the BD LSRII flow cytometer and the data was analyzed by FlowJo single cell analysis software.

### Measurement of blood cells after total body irradiation (TBI)

Recovery of blood cells was monitored after TBI by analyzing blood samples from irradiated mice with an HEMAVet 950FS automatic veterinary hematology analyzer (Drew Scientific, INC, France). Blood was obtained from live mice by submandibular bleeding, which enabled sequential measurements over time on the same mouse [37].

### Immunoblotting

Cells were lysed in Cell Signaling lysis buffer (#9803) containing 1mM PMSF (Sigma P7626), 2mM NaF (Sigma S-1504), 2mM Na_3_VO_4_ (Sigma S-6508) and 1x protease inhibitor cocktail (Roche 5892953001). 50μg total protein/sample of total lysate was subjected to sodium dodecyl sulfate polyacrylamide gel electrophoresis. Separated proteins were transferred to polyvinylidene difluoride membranes (EMD Millipore IPVH00010). The membranes were then probed with appropriate primary antibodies, followed by horseradish peroxidase-conjugated secondary antibodies. The blots were developed with HyGLO chemiluminescence reagent (Denville Scientific #E2400) and exposed to HyBlot CL autoradiography film (Denville Scientific #E3012), which was scanned with a flat-bed scanner (UMAX Technologies, Hsinchu, Taiwan). Band densitometry analysis was performed using the NIH ImageJ program. Protein expression was normalized to either Glyceraldehyde 3-phosphate dehydrogenase (GAPDH), (Cell Signaling, #2118S) or β-actin (Sigma #A5441) expression.

### Quantitative real-time PCR (qRT-PCR)

Total RNA was extracted from BM-MNCs using TRIzol^R^ reagent (LifeTechnologies #15596–018) according to the manufacturer’s instructions. cDNA was synthesized from total RNA with qScript reverse transcriptase (Quanta Biosciences #95048–100) using random and oligo(dT) primers as per manufacturer’s protocol. The StepOnePlus™ Real-Time PCR thermal cycling instrument (Invitrogen, Carlsbad, CA) was used with the iTaq^TM^ universal SYBR^R^ green fluorescent supermix (Biorad #172–5121) to quantify mRNA expression of p53 in a one-step reaction following manufacturer’s instructions. GAPDH was used as the housekeeping gene. Primer sequences used were as follows; p53 forward, 5’-TATGTGCACGTACTCTACTCTCCTC-3’; p53 reverse, 5’-TGCTGTGACTTCTTGTAGATG-3’; GAPDH forward, 5’-ACCACAGTCCATGCCATCAC-3’; GAPDH reverse, 5’-CACCACCCTGTTGCTGTAGCC-3’ (IDT Technologies). Specificity of the PCR reactions was confirmed by melting curves. p53 mRNA expression was normalized to the relative amount of GAPDH expression.

### γ-H2AX foci detection by immunocytofluorescence

Alexa Fluor 488 conjugated anti-γ-H2AX (Ser139) antibody was used to detect DNA DSBs following the manufacturer’s protocol. BM-MNCs were exposed to 6 Gy irradiation. Irradiated cells were fixed 4 Hrs post exposure, blocked for 1 Hr in cell signaling blocking buffer and stained with the anti- γ-H2AX (Ser139) antibody (1:1000) or rabbit IgG isotype control (0.025μg/ml) overnight at 4°C. After three rinses with phosphate buffered saline (PBS), the cells then stained with DAPI (Life Technologies, #D1306) for 15 min at room temperature. Prolong^R^ Gold Anti-Fade Reagent (Life Technologies, #P36930) was used to mount the cells after washing the cells following DAPI staining. Slides were viewed and pictures taken on a FV1000 v1.5 confocal microscope (Olympus, Shinjuku, Tokyo, Japan).

### Statistics

Statistical significance of differences between groups was evaluated by Student’s t test or Tukey’s multiple comparisons test as appropriate and p values < 0.05 were considered significant.

## Results

### Radiation induces a similar increase in apoptosis of *Egr1*^*+/+*^ and *Egr1*^*-/-*^ primary bone marrow cells *in vitro*

**Fig 1:**
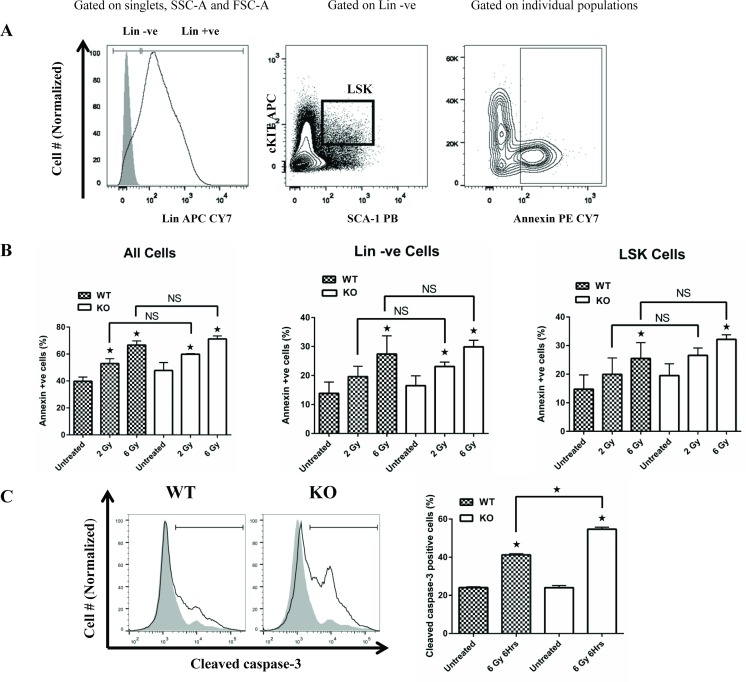
Ionizing radiation induced apoptosis of primary *Egr1*^*+/+*^ and *Egr1*^-/-^ BM-MNCs equally in vitro. **(A)** Representative analysis of irradiated lineage negative (Lin–ve) enriched BM-MNCs by flow cytometry. BM-MNCs isolated from WT and *Egr-1* KO mice were enriched for Lin–ve cells (as described in the methods). After 2 days of cytokine stimulation (mSCF-50ng/ml, mIL3-10ng/ml, hIL6-10ng/ml), Lin–ve enriched BM-MNCs were left untreated, or exposed to 2 Gy or 6 Gy irradiation (1 × 10^6^/ml). 24Hrs after irradiation, cells were stained with c-KIT-APC, Sca-1-PB and streptavidin APC CY7 antibodies, and annexin-V-PE CY7 to identify LSK and apoptotic cells respectively. A minimum of 500,000 cells were collected per sample on the BD LSR II flow cytometer and the data was analyzed using the FlowJo single cell analysis software for the percentage of apoptotic cells (by annexin) in the various cell populations (all cells, lin–ve cells and LSK cells). **(B)** Annexin +ve cells in the BM-MNC, and gated subpopulations from WT and *Egr-*1 KO mice are shown. **(C)** BM-MNC were stained for cleaved caspase-3. Left panel shows representative flow cytometry profile of irradiated WT or KO BM-MNCs (clear histograms) overlaid on untreated BM-MNCs (gray histograms). Summary of data from triplicate cultures is shown in the right panel. Results represent mean ± SE of triplicate cultures. *Indicates p<0.05 comparing untreated cells to cells exposed to radiation. Results from one of two experiments with similar outcomes are shown.

Myeloid malignancies arise from hematopoietic stem or progenitor cells and expansion of myeloid blasts drives progression [1, 38]. Radiation and chemotherapy treatments used to treat many types of cancer induce apoptosis in the bone marrow stem cell compartment. EGR1 has been shown to be important for apoptosis induction in several model systems and is one of the deleted genes located in the CDR of t-MNs with del (5q). To determine the role of EGR1 in apoptosis of bone marrow cells, *Egr1*^*+/+*^ (wildtype) and *Egr1*^*-/-*^ (knockout) bone marrow mononuclear cells (BM-MNCs) enriched for primitive cells by depletion of mature lymphoid and myeloid cells were exposed to ionizing radiation (2 Gy and 6 Gy) and apoptosis was assessed by annexin-V staining 24Hrs post radiation. LIN-ve cells were defined by the absence of cell surface expression of mature differentiation markers while stem cells were defined by double positive expression of both Sca-1 (stem cell antigen-1) and c-KIT on lineage negative cells (LSK) as illustrated in Fig 1A. Radiation significantly increased apoptosis of both wildtype and knockout BM-MNCs (Fig 1B). We observed that *Egr1*^*-/-*^ both lineage negative and LSK cells underwent radiation induced apoptosis as well as their *Egr1*^*+/+*^ counterparts (Fig 1B). Consistent with the annexin assay, we observed a significant increase in the activation of caspase-3 by wildtype and *Egr1* knockout BM-MNCs 6 Hrs post radiation exposure (Fig 1C). Although the percentage of cells with radiation induced activated caspase-3 was slightly higher for *Egr1*^*-/-*^ BM-MNCs compared to *Egr1*^*+/ +*^ (p<0.05) (Fig 1C), this difference did not translate to a statistically significant increase in annexin positive cells (p>0.05) (Fig 1B). The differing outcomes could be due to the timing of two assays, caspase activation being measured at six hours and annexin expression at 24 hours. These data suggest that EGR1 is not required for radiation-induced apoptosis of murine bone marrow cells.

### *Egr1*^*+/+*^ and *Egr1*^*-/-*^ mice recover equally well after exposure to a sub-lethal dose (6.5 Gy) of total body irradiation (TBI)

**Fig 2:**
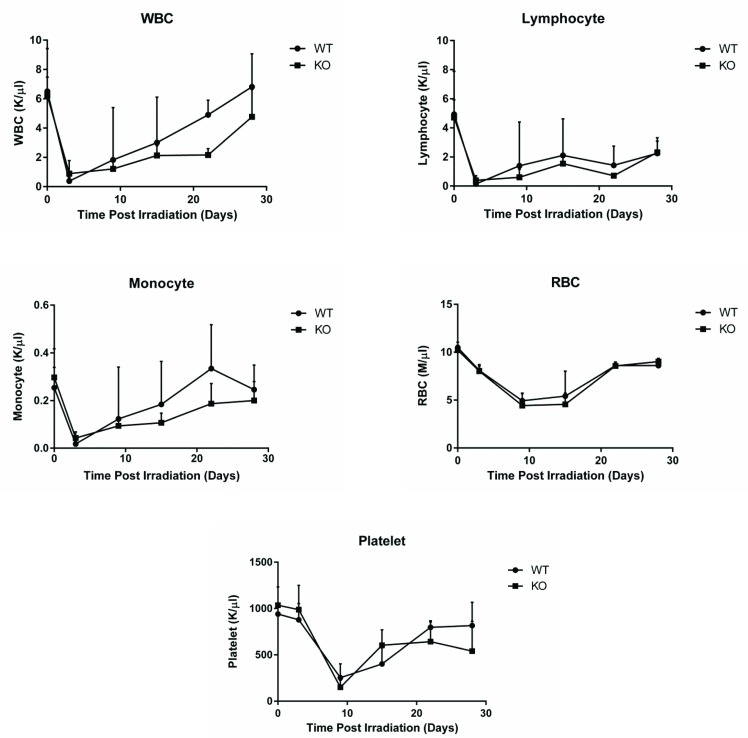
*Egr1*^*+/+*^ and *Egr1*^-/-^ mice have comparable recovery kinetics of blood cells after sub-lethal TBI. Mice were exposed to 6.5 Gy TBI. Blood cells were enumerated at 3, 9, 15, 22, and 28 days after TBI by the HEMAVet 950FS automatic veterinary hematology analyzer. Baseline (day 0) measurement was done before irradiation. The results are expressed as the mean of blood cells concentration ± SE (n = 8 mice/ group). Results from one of two similar experiments are shown.

We next evaluated if EGR1 contributed to radiation induced cell death in an *in vivo* system. It has been previously shown that BM-MNCs of wildtype (WT) mice recover almost completely to normal levels by 28 days post sub-lethal (6.5 Gy) TBI [39]. We investigated whether EGR1 improved or delayed this recovery by monitoring peripheral blood numbers of different cell types in *Egr1*^*+/+*^ and *Egr1*^*-/-*^ mice exposed to 6.5 Gy TBI. There was no statistically significant difference between *Egr1*^*+/+*^ and *Egr1*^*-/-*^ mice in the recovery of white blood cells, lymphocytes, monocytes, red blood cells (RBC) and platelets during the 28 days monitoring period after 6.5 Gy TBI (Fig 2). These results are in agreement with the *in vitro* data suggesting that EGR1 does not play a significant role in radiation-induced apoptosis of hematopoietic progenitors and stem cells in the bone marrow.

### DNA double strand break damage upregulates p53 protein levels in *Egr1*^*+/+*^ and *Egr1*^*-/-*^ BM-MNCs

**Fig 3:**
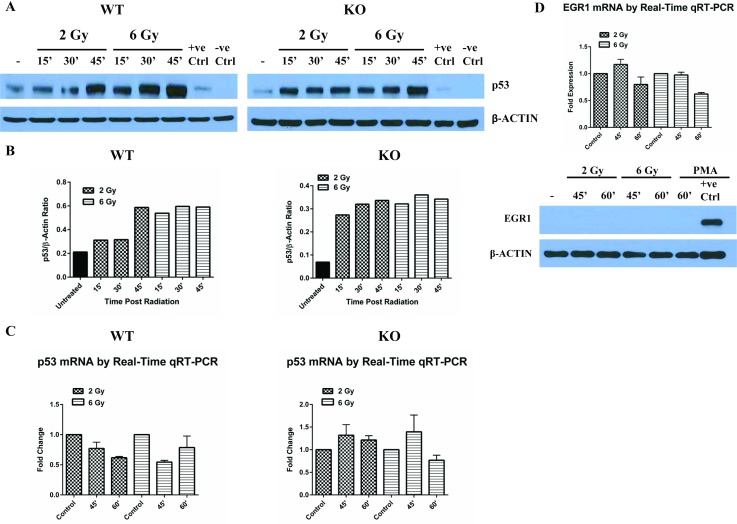
Increased p53 protein expression in irradiated *Egr1*^*+/+*^ and *Egr1*^-/-^ BM-MNCs is independent of mRNA expression. **(A)** Cell lysates from irradiated Lin–ve enriched BM-MNCs (2 Gy or 6 Gy) at different time points (15’, 30’ and 45’) after radiation exposure were used for immunoblotting for p53. A549 and H358 cell lysates were used as positive and negative controls respectively. (**B)** Intensities of p53 bands were normalized to β-actin. Values are expressed as densitometric ratios. (**C)** BM-MNCs were lysed 45’ or 60’ after irradiation (2 Gy or 6 Gy). The RNA extracted from these lysed cells was used to determine p53 mRNA expression by qRT-PCR. (**D**) Cell lysates from phorbol 12-myristate 13-acetate (PMA) stimulated (30ng/ml) or irradiated Lin–ve encriched *Egr1*^*+/+*^ BM-MNCs (2 Gy or 6 Gy) at different time points (60’) and (45’, 60’) respectively. Lysate of normal splenic wildtype B cells stimulated with PMA (30ng/ml) for 60’ was used as a positive control for EGR1 expression [41]. RNA extracted from wildtype BM-MNCs in (C) was used to determine EGR1 mRNA expression by qRT-PCR.

EGR1 was shown to be required for radiation induced apoptosis of several cell types by virtue of its ability to induce upregulation of p53 [27, 28, 33]. Hence we evaluated if radiation induced increase in p53 levels in BM-MNCs was dependent on EGR1. Wildtype and *Egr1* knockout BM-MNCs were exposed to two different doses of ionizing radiation (2 Gy and 6 Gy) and p53 protein and mRNA expression were determined by western blot and qRT-PCR respectively. Radiation rapidly upregulated p53 protein levels in both *Egr1*^*+/+*^ and *Egr1*^*-/-*^ BM-MNCs (Fig 3A and 3B) even in the absence of significant upregulation of p53 mRNA (Fig 3C). These results suggest radiation induced increase in p53 protein levels in BM-MNCs is independent of EGR1. Accordingly, we found that neither EGR1 protein nor mRNA expression in *Egr1*^*+/+*^ BM-MNCs was significantly changed by exposure to radiation (Fig 3D). In fact, EGR1 protein was undetectable in irradiated BM-MNCs using same conditions for which it was readily detectable in phorbol 12-myrsitate 13-acetate (PMA) stimulated normal splenic B cells. This radiation response of BM-MNCs is unlike that of mouse embryonic fibroblasts (MEFs) where radiation induced increase in p53 protein and subsequent apoptosis is EGR1 dependent [27]. Increase in p53 protein by ionizing radiation that is independent of transcription can be due to activation of the DNA damage response pathway. Ionizing radiation induces more of the deleterious DNA DSBs than single strand breaks [40].

DNA DSBs induce apoptosis through a signaling cascade that is triggered by recruitment and activation of DNA damage-responsive kinase, ataxia telangiectasia mutated (ATM) [42]. Activated ATM phosphorylates several substrates, its mediator check point kinase 2 (chk2) and histone H2AX included. Chk2 relieves p53 from Mdm2 directed proteasomal degradation by phosphorylating it at serine 15 (Ser15), hence stabilizing p53 protein levels [42, 43]. Phosphorylated histone H2AX (γ-H2AX) spreads the damage signal along the chromatin making it an acceptable marker for DNA DSBs [44].

**Fig 4:**
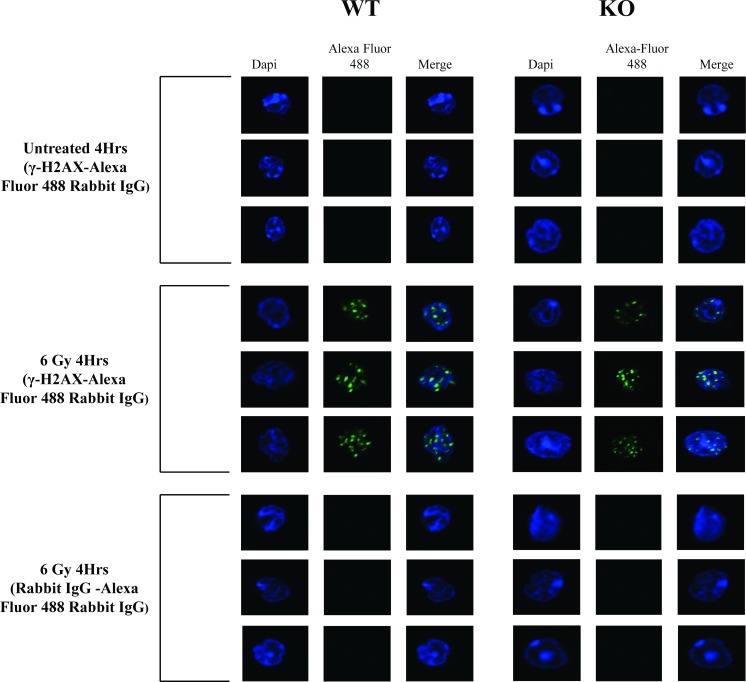
Radiation induces DNA DSBs in *Egr1*^*+/+*^ and *Egr1*^-/-^ BM-MNCs. Immunofluorescence analysis of irradiated BM-MNCs for γ-H2AX foci was performed as described in the methods. Representative immunofluorescence images of γ-H2AX foci in BM-MNCs 4Hrs after exposure to 6 Gy irradiation.

**Fig 5:**
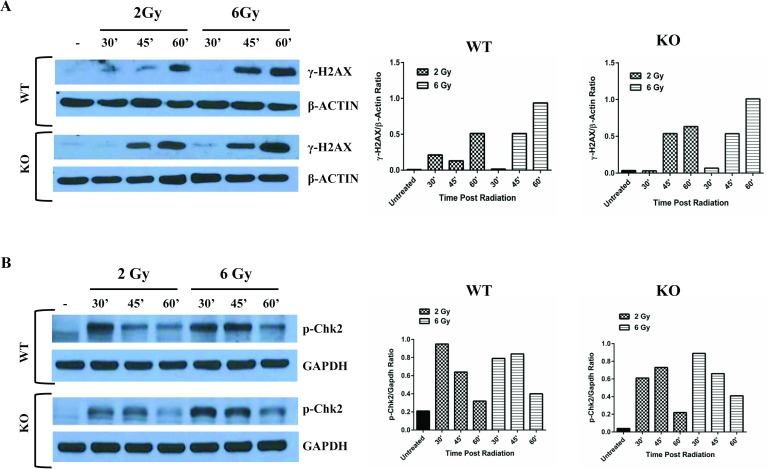
Radiation activates the DSB DNA response pathway in *Egr1*^*+/+*^ and *Egr1*^-/-^ BM-MNCs. **(A)** Left: γ-H2AX protein expression by immunoblotting in irradiated BM-MNCs 30’, 45’ and 60’ after radiation exposure (2 Gy or 6 Gy). Right: Intensities of γ-H2AX bands were normalized to β-actin and are expressed as densitometric ratios. **(B)** Left: p-Chk2 protein expression by immunoblotting in irradiated BM-MNCs at different times after radiation exposure (2 Gy or 6 Gy). Right: p-Chk2 band intensities were normalized to those of GAPDH and are expressed as densitometric ratios. Results are representative of two experiments.

We next evaluated if radiation induced DSBs to a similar extent in Egr1^+/+^ and Egr1^-/-^ BM-MNCs. Immunofluorescence analysis showed that radiation induced γ-H2AX foci in BM-MNCs from *Egr1*^*+/+*^ and *Egr1*^*-/-*^ mice equally well (Fig 4). Furthermore, immunoblotting also showed a similar increase of γ-H2AX in irradiated Egr1^+/+^ and Egr1^-/-^ BM-MNCs (Fig 5A). In line with this, we investigated if radiation induced ATM activation by evaluating the levels of phosphorylated Chk2 in irradiated *Egr1*^*+/+*^ and *Egr1*^*-/-*^ BM-MNCs. Shown in Fig 5B, radiation clearly increased ATM activity of both wildtype and knockout BM-MNCs to a similar extent, as reflected by increased levels of phospho-Chk2.

## Discussion

EGR1 is zinc finger transcription factor located in the CDR of chromosome 5q [4, 7]. Several studies have demonstrated that it is a tumor suppressor in several model systems, human malignancies included [29, 45–48]. Haploinsufficiency of EGR1 cooperates with other mutations in the development of murine myeloid neoplasms [19, 26, 49], suggesting it might play a significant role in malignant transformation of stem cells leading to t-MNs. The ability of EGR1 to maintain HSC quiescence and retention in the bone marrow niche [22] suggests hyperproliferation of stem cells is a plausible mechanism by which haploinsufficiency of this protein could promote malignant transformation in t-MNs. EGR1 regulates cell growth and apoptosis in part by regulating expression of p53 [25, 27, 33, 34, 47]. In normal murine fibroblasts, normal hippocampal neurons, melanoma cells, a variety of human tumor cell lines, gliobastoma, non-small cell lung cancer, breast carcinoma, skin cancer as well as head and neck cancer, EGR1 is either pro-apoptotic or suppresses growth [27–29, 33, 34, 47, 48, 50–53]. On the contrary, in pancreatic beta cells, glioma and colorectal cancer cells, prostate cancer, B-cell lymphoma and normal B cells EGR1 has anti-apoptotic or pro-proliferative role [25, 28, 30, 41, 54–57]. Decreased expression of EGR1 could lead to resistance to apoptosis of stem cells and may possibly contribute to malignant transformation in t-MNs. Presently, it is unknown if EGR1 has a pro- or anti-apoptotic role in radiation induced apoptosis of BM cells. We investigated this never before studied possibility by utilizing radiation induced apoptosis as a tool to evaluate the role of EGR1 in apoptosis of bone marrow cells. Focus on the bone marrow was imperative because myeloid malignancies arise from this tissue [1]. Interestingly, we found that EGR1was entirely expendable for the radiation induced apoptotic response of murine bone marrow lineage negative cells that contain LSK cells which are comprised of multipotent progenitor, short term, and long term hematopoietic stem cells. Furthermore, unlike in many other cell types and some cancers [27, 34, 47], p53 induction in murine bone marrow cells by radiation was surprisingly independent of EGR1.

The aforementioned results, though unexpected are not unusual as they highlight the complexity of biological systems. An indication to such complexity as it pertains to EGR1 regulation and its downstream effects is highlighted by the fact that while it is thought to be a tumor suppressor in most malignancies, EGR1 seems to be surprisingly oncogenic in prostate cancer [25, 54, 55]. This observation parallels the evidence suggesting that it can be a positive [27–29, 33, 34, 47, 48, 50–53] or a negative [25, 28, 30, 41, 54–57] regulator of apoptosis in different systems. Instead, we found that EGR1 is neither a positive nor a negative regulator of radiation induced apoptosis in murine bone marrow cells. There is evidence suggesting that EGR1 can regulate radiation induced p53 by stabilizing the p53 protein [27] as well as increasing its transcription [34, 47]. We investigated this question and found that irradiation does not increase p53 mRNA in EGR1 sufficient as well as deficient BM cells. Rather, we found that phosphorylation of Chk2 and histone H2AX was increased in both wild type and EGR1 null bone marrow cells subjected to radiation. Chk2 is known to phosphorylate Mdm2 and prevent its ability to target p53 for proteasome mediated degradation. Accordingly, radiation induced increase in p53 protein in murine bone marrow cells is likely achieved by protein stabilization through DSB induced DNA repair response mechanisms that are independent of EGR1. These results are important in the context of the findings that incidence of t-MNs is on the rise as the number of cancer survivors at risk increases with improvements in cancer treatments [49]. The prognosis of t-MNs is generally very poor with a median survival time ranging from 9–36 months and median remission duration of just a few months [6].

In summary, radiation appears to induce DNA damage and Chk2 phosphorylation in bone marrow mononuclear cells leading to p53 activation and apoptosis. The data presented here indicate that EGR1 may not significantly regulate the aberrant apoptosis response of hematopoietic stem and progenitor cells in myeloid malignancies. Nevertheless, it plays a significant role in regulating HSC quiescence and has been shown to promote development of murine myeloid neoplasms [19, 22, 26, 49]. Hence the observation that 5q chromosome aberration that includes loss of one *Egr1* allele is associated with t-MNs [6, 58] suggests that haploinsufficiency of EGR1 may cooperate with other mutations in the development of myeloid malignancies.
